# Multiswitchable photoacid–hydroxyflavylium–polyelectrolyte nano-assemblies

**DOI:** 10.3762/bjoc.17.17

**Published:** 2021-01-19

**Authors:** Alexander Zika, Franziska Gröhn

**Affiliations:** 1 Department of Chemistry and Pharmacy & Interdisciplinary Center for Molecular Materials (ICMM) and Bavarian Polymer Institute (BPI), Friedrich-Alexander-Universität Erlangen-Nürnberg, Egerlandstr. 3, D-91058 Erlangen, Germany

**Keywords:** electrostatic self-assembly, hydroxyflavylium, multiswitchable, photoacid, polyelectrolyte

## Abstract

Light- and pH-responsive nano-assemblies with switchable size and structure are formed by the association of a photoacid, anthocyanidin, and a linear polyelectrolyte in aqueous solution. Specifically, anionic disulfonated naphthol derivatives, neutral hydroxyflavylium, and cationic poly(allylamine) are used as building blocks for the ternary electrostatic self-assembly, forming well-defined supramolecular assemblies with tunable sizes of 50 to 500 nm. Due to the network of possible chemical reactions for the anthocyanidin and the excited-state dissociation of the photoacid upon irradiation, different ways to alter the ternary system through external triggering are accessible. The structure and trigger effects can be controlled through the component ratios of the samples. Dynamic and static light scattering (DLS, SLS) and ζ-potential measurements were applied to study the size and the stability of the particles, and information on the molecular structure was gained by UV–vis spectroscopy. Isothermal titration calorimetry (ITC) provided information on the thermodynamics and interaction forces in the supramolecular assembly formation.

## Introduction

Supramolecular nanoscale assemblies responding to multiple stimuli are highly desirable in various fields including transport systems, sensors, and optoelectronic applications [1–8]. Self-assembly into supramolecular structures and molecular recognition processes have been established based on different non-covalent interactions such as hydrogen bonding [9–12], π–π interaction [13–17], hydrophobic effects, and amphiphilicity [18–21]. It has been highly desirable to establish concepts that could be applied in aqueous solution, with the Schmuck binding motif – the guanidiniocarbonyl‐pyrrole zwitterion binding motif – representing a most versatile binding motif with potential from nanostructure design to biomedicine [22–25]. In addition, it is desirable to establish general concepts that do not rely on specific binding motifs. Here, electrostatic self-assembly was shown to yield a variety of supramolecular nanostructures [4,26–33]. We have built the nanoscale assemblies from polyelectrolytes and multiply oppositely charged molecules based on electrostatic interactions and secondary interactions such as π–π stacking [34–40]. The size and shape could be tuned through the free energy and the enthalpy/entropy interplay in the assembly process, which again are encoded in the molecular building block structure [31,37].

Supramolecular structures can respond to external triggers including pH change [41–43], light [44–50], electrochemical stimuli [51–52], and temperature [53–54]. Of particular interest is the responsivity to light due to its non-invasiveness, which can be achieved with molecules that undergo photodimerization [55–57], photocleavage [58–59], and *cis–trans* photoisomerization [60–65]. While it had been well-established to access oligomer formation and gelation, a light-switchable particle size has remained a challenge until realized through the mentioned electrostatic self-assembly combining a polyelectrolyte and an ionic azo dye [4]. Moreover, recently we have developed a switchable nano-assembly system based on photoacids [66–67]. Photoacids are molecules for which light irradiation leads to an enhanced proton dissociation and thus to a more highly charged molecule. Furthermore, systems which respond to more than one external trigger have become of great interest [68–73].

**Scheme 1:**
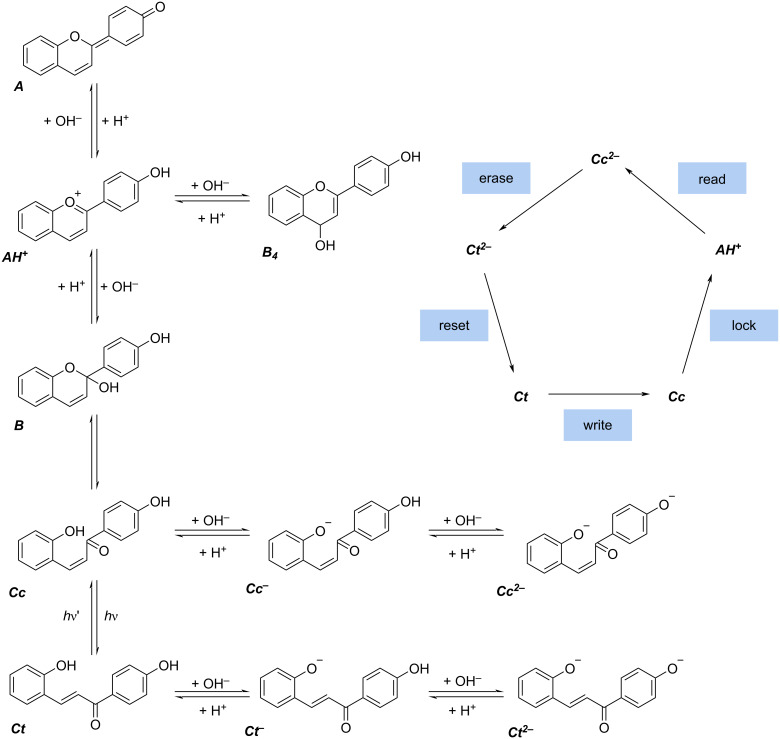
The chemical network of reactions for 4-hydroxyflavylium (left) and the write-lock-erase cycle (right) [74–75].

Hydroxyflavylium cations can exhibit a network of different chemical reactions enabling these molecules to perform as a molecular level optical memory [74–78]. The network of reactions is displayed in Scheme 1 for 4-hydroxyflavylium. With a “write-lock-read-unlock-erase” cycle it can serve as an optical memory system with multiple storage. In this process, information is photochemically written in form of ***Cc*** and locked by a chemical input as ***AH^+^***, so that the information is protected and can be read by spectroscopy. The storage can be unlocked by conversion to the ***Cc^2−^*** form, and then be erased by another photon or thermal energy through the reaction to ***Ct^2^***^−^, and lastly it can be reset by a chemical input, completing the cycle. A number of studies exist that focused on the understanding and variation of this cycle by synthesizing different derivatives of 4-hydroxyflavylium. Yet, hydroxyflavylium molecules have not been exploited in self-assembly to form water-compatible switchable supramolecular nanostructures that may be responsive to different external triggers such as light, pH, and temperature.

**Scheme 2:**
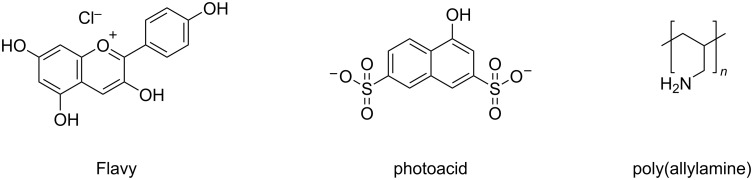
The building blocks used for the self-assembly in this study: pelargonidin chloride (Flavy), 1-naphthol-3,6-disulfonate (1N36S, photoacid), and poly(allylamine). The photoacid hydroxy group acidity increases upon photoexcitation. Flavy can undergo different reactions as given in Scheme 1. Poly(allylamine) is present in the protonated form as a cationic polyelectrolyte in neural to acidic solution.

**Scheme 3:**
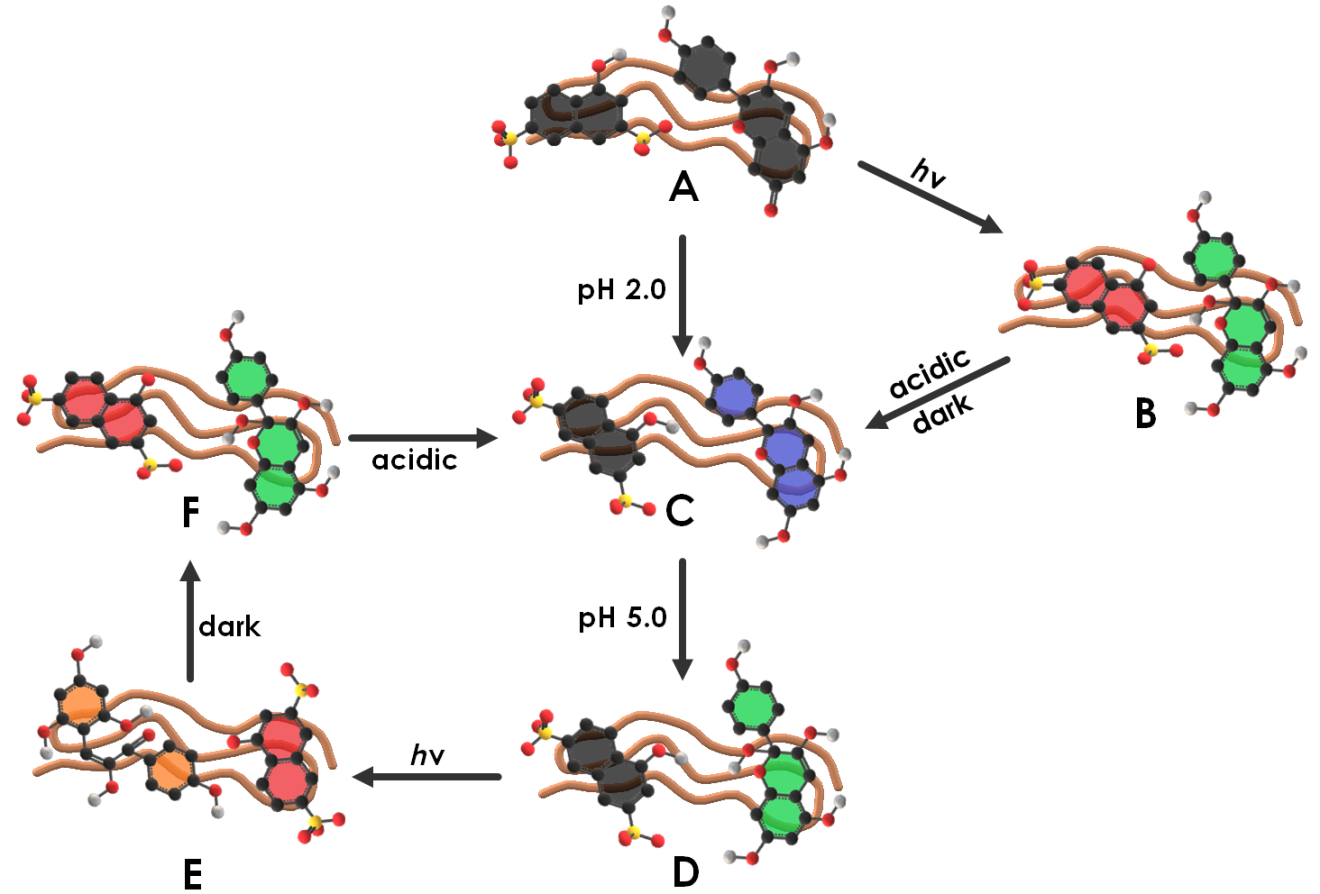
Overview of the different states of the multi-switchable system consisting of Flavy, 1N36S, and poly(allylamine) (simplified schematic representation displaying one molecule of each component per assembly only). **A**: The protonated photoacid and Flavy in form of ***A***. **B**: The deprotonated photoacid and Flavy in form of ***B****.* **C**: The protonated photoacid and Flavy in form of ***AH^+^***. **D**: The protonated photoacid and Flavy in form of ***B***. **E**: The deprotonated photoacid and Flavy in form of ***Cc***. **F**: The deprotonated photoacid and Flavy in form of ***B***.

In this respect, it is highly desirable to establish novel supramolecular nanoscale structures in aqueous solution that can respond to multiple triggers. Herein, we studied a multi-responsive system based on the self-assembly of a cationic polyelectrolyte and two organic molecules in aqueous solution. The building blocks pelargonidin chloride (Flavy), 1-naphthol-3,6-disulfonate (1N36S), and poly(allylamine) are depicted in Scheme 2. The aim was to exploit the photoacid's unique capability to undergo photoinduced intermolecular excited state proton transfer reactions, both to create a more highly charged photoacid molecule – in this case changing from dianionic to trianionic – and to affect the pH-dependent Flavy molecule and thereby the properties of the nano-assembly. In doing so, this system offers multiple interconnected possibilities of triggering molecular and nanoscale structure, as indicated in Scheme 3.

To gain fundamental understanding on these possibilities, different trigger paths and the dependency on the assembly composition were studied with regard to the effect on the nano-assembly characteristics by dynamic and static light scattering, UV–vis spectroscopy, and isothermal titration calorimetry.

## Results and Discussion

### Characterization of the flavy building block

**Figure 1:**
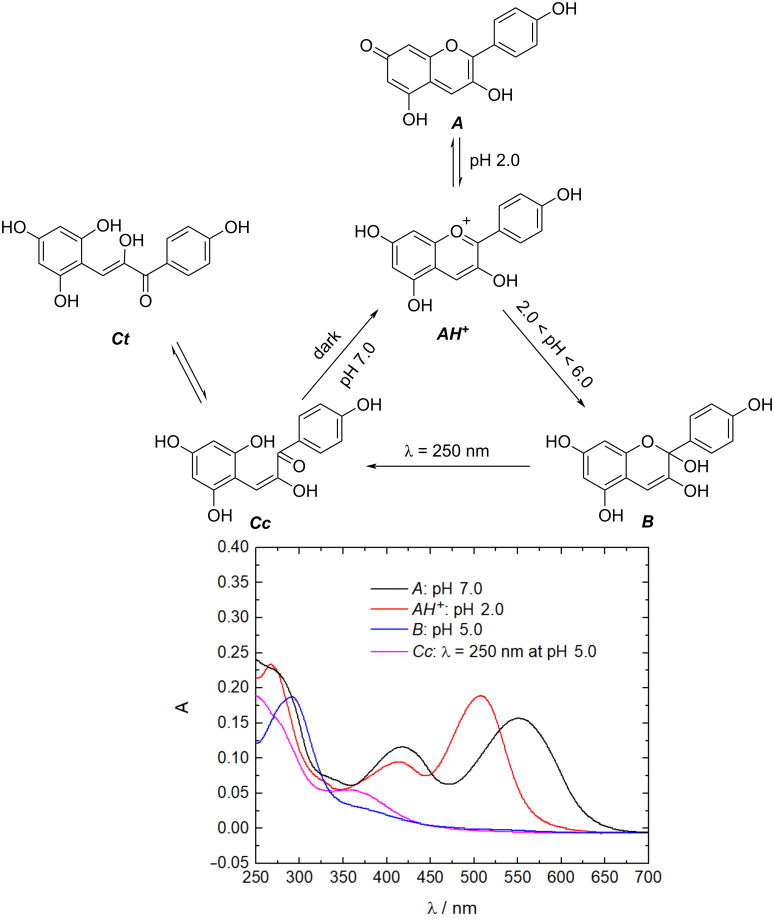
Top: pelargonidin cation (Flavy) and network of chemical reactions; bottom: corresponding UV–vis spectra of the different states of Flavy.

In the first step the switchability and the photostability of pelargonidin chloride (Flavy) were investigated. The switching ability of Flavy is already well-known [79–80]. Here it was followed with UV–vis spectroscopy to monitor the differences between the states. As can be seen in Figure 1, the different forms of Flavy vary in the light absorption. While the forms ***A*** and ***AH^+^*** show an absorption at λ = 500 and λ = 550 nm, respectively, due to their expanded electronic system, the absorption maximum of form ***B*** lies in the UV region at λ = 290 nm. The open forms ***Cc*** and ***Ct*** cannot be separated and show an absorption at λ = 360 nm.

**Figure 2:**
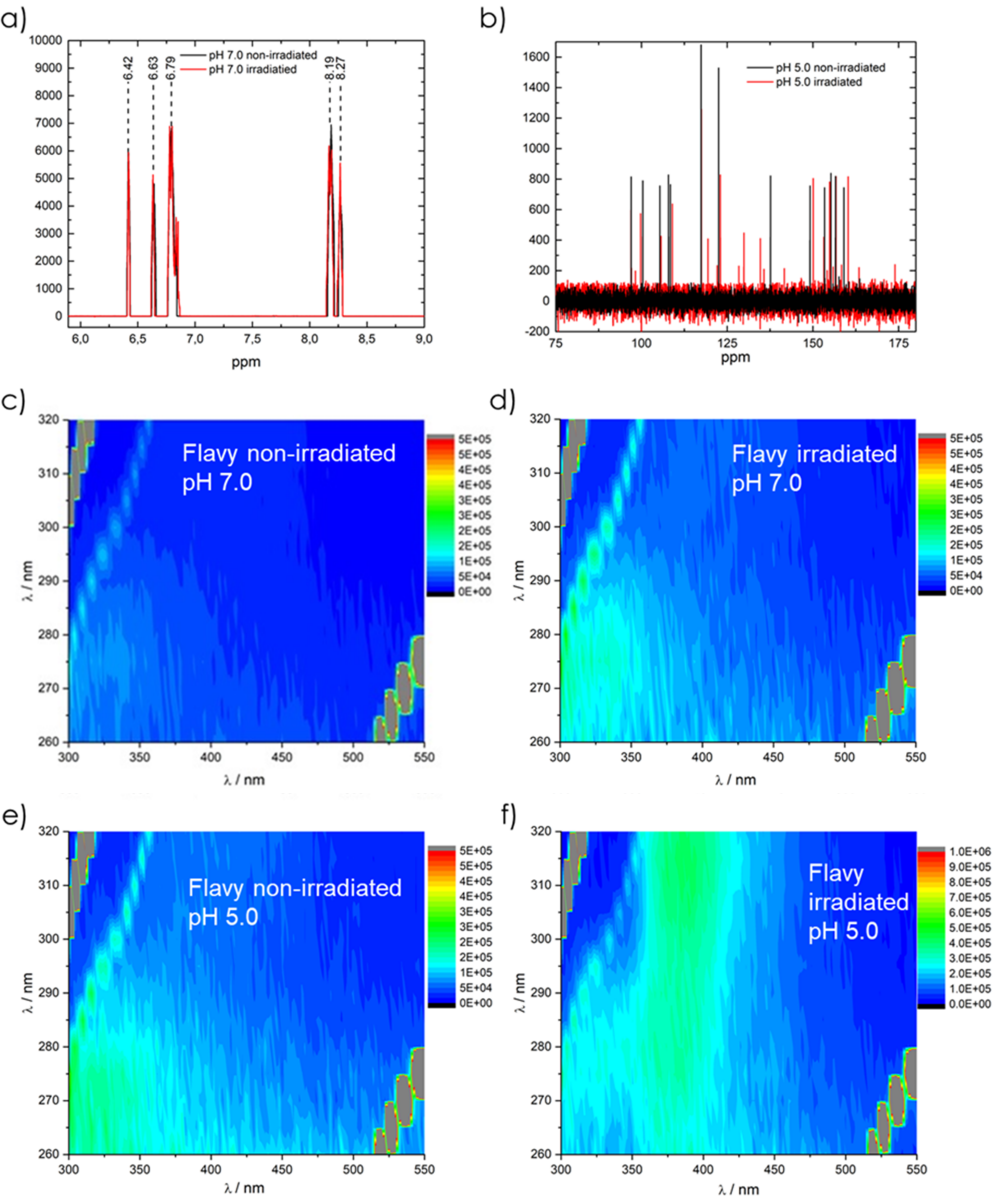
Characterization of Flavy: a) ^1^H NMR spectrum at pH 7.0 (form ***A***) before and after irradiation; b) ^13^C NMR spectrum at pH 5.0 (form ***B***) before and after irradiation; c) and d) 3D fluorescence spectroscopy analysis at pH 7.0 (form ***A***) before (c) and after (d) irradiation; e) and f) fluorescence spectroscopy analysis of Flavy at pH 5.0 (form ***B***) before (e) and after (f) irradiation.

Since the aim of this study was to develop a multiswitchable system that acts under light irradiation, it is also of importance that the Flavy molecule is stable regarding the irradiation at λ = 302 nm. Therefore, the Flavy molecule was irradiated at form ***A*** and ***B*** and then analyzed by ^1^H, ^13^C NMR, and fluorescence spectroscopy, as given in Figure 2. The ^1^H NMR measurements show that the irradiation of state **A** does not lead to any changes. The fluorescence spectrum shows a small transformation to form ***B***. The relatively weak intensity suggests that this was not due to the irradiation but the well-known hydrolysis. In case of form ***B***, the ^13^C NMR data show that the irradiation leads to form ***Cc***, which can be seen from the new peaks at 127 ppm, 134 ppm, and 175 ppm, respectively. Furthermore, a third form appears to be present, even though the concentration of this state is a lot smaller. Due to the signals at 141 ppm and 164 ppm the third form likely represents the Flavy molecule that is hydrolyzed at position 4 instead of 2. The fluorescence spectrum also shows the transformation of ***B*** to ***Cc*** with the fluorescence band changing from λ = 300 nm to λ = 400 nm.

### Formation and switching cycles of the ternary assemblies

In the first step, the supramolecular assemblies were formed by mixing the photoacid 1N36S with Flavy and adding the cationic polyelectrolyte poly(allylamine) at pH 7.0. At this pH value, both the polyelectrolyte and also the photoacid are protonated [81–82]. Upon irradiation, the p*K*_a_ of the photoacid decreases to p*K*_a_ = −2.6 leading to deprotonation [82]. In addition to the electrostatic self-assembly of the photoacid and the polyelectrolyte which can be altered through irradiation, the Flavy molecule may associate with the polyelectrolyte via hydrogen bonding and dipolar interaction, which will also respond to the pH value, either by a direct pH change or via the effect of the photoexcitation of the photoacid. To state the assembly composition when studying these effects, two ratios were introduced. The loading ratio *l* describes the small molecule to polyelectrolyte stoichiometry: It is defined as the molar concentration of the negative charges of the photoacid plus the molar concentration of formal Flavy binding sites – formally set to 4, independent of the pH value – divided by the molar concentration of the cationic polyelectrolyte charges (Equation 1). With this, the loading ratio characterizes the composition of the sample and keeps the value when the pH is changed (4 refers to the number of OH groups in the ***AH^+^*** state). The concentration ratio *k* represents the relative molar concentration of the photoacid and the Flavy molecule, as given in Equation 2:

[1] $$l=\frac{z^{-}\times c(\text{1N36S})+\text{ binding sites Flavy}\times c(\text{Flavy})}{z^{+}\times c(\text{polyallylamine)}}$$

[2] $$k=\frac{c\text{(1N36S)}}{c\text{(Flavy)}}$$

**Scheme 4:**
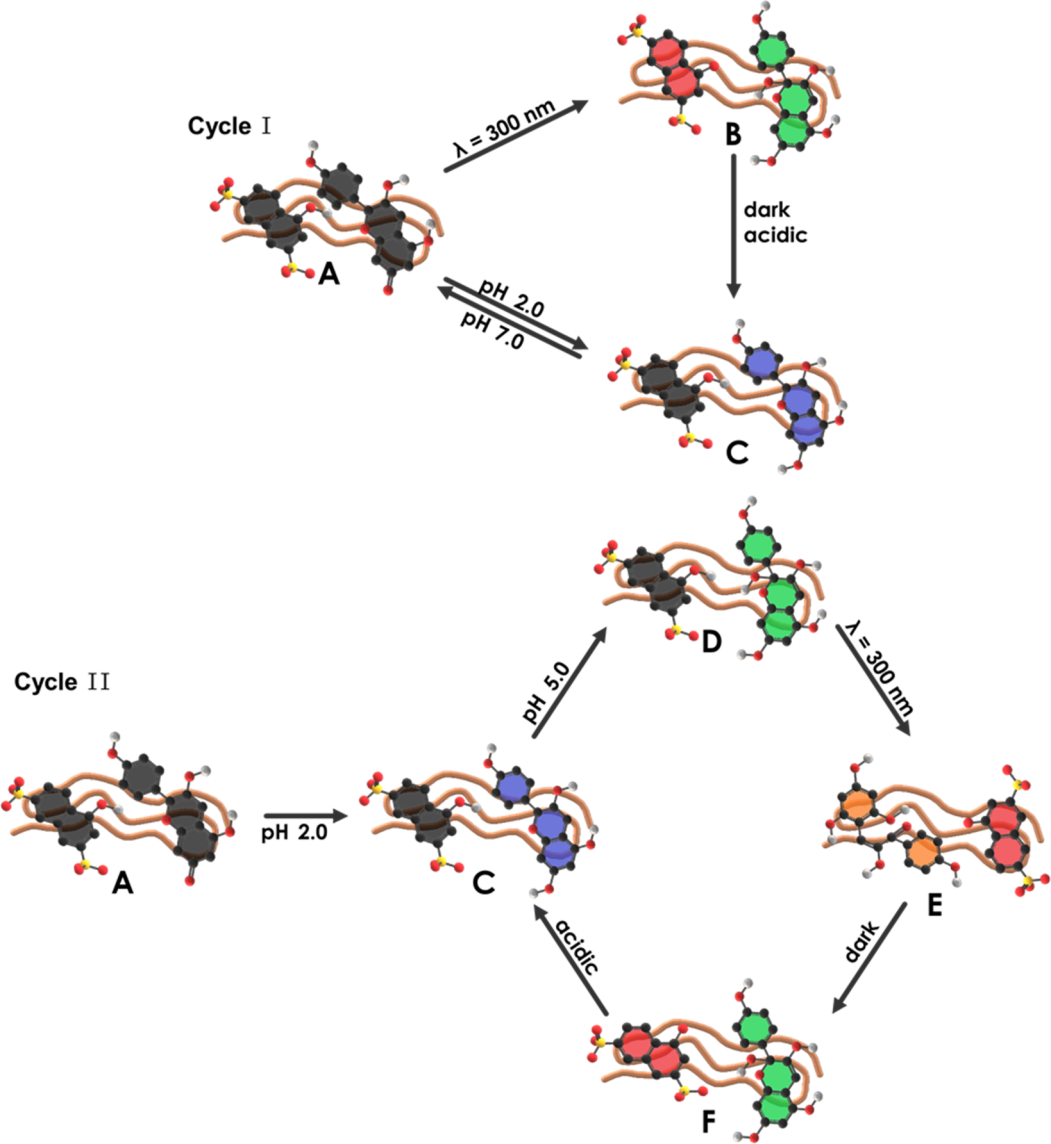
Overview of the different states of the two main cycles switching the system consisting of 1N36S, Flavy, and poly(allylamine).

First, a system with a loading ratio of *l* = 0.9 and a concentration ratio of *k* = 1.0 was investigated. In this case, we focused on the two main switching cycles displayed in Scheme 4. Cycle I starts with the excitation of the photoacid by irradiation at λ = 300 nm with the aim to achieve the protonation of the hydroxyflavylium molecule, and the subsequent opening to the ***Cc*** form. After keeping the assemblies in the dark and addition of HCl, assemblies as in state **C** should result. Compared to that, cycle II starts with the switching of the Flavy molecule from **A** to **D** over **C**. The excitation of the photoacid should lead to the open form ***Cc*** in state **E**. Again, keeping the assemblies in the dark and subsequently adding HCl should reform the same structures as in the beginning.

**Figure 3:**
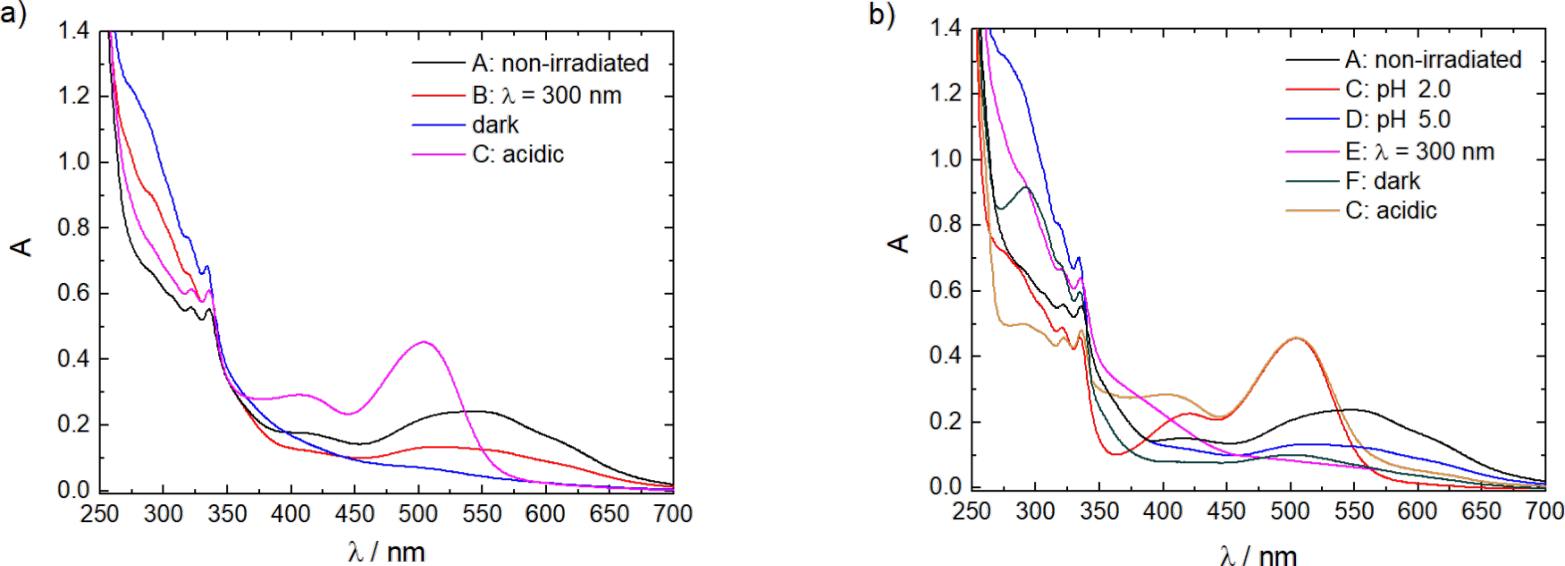
UV–vis spectroscopy of the ternary nano-assemblies for cycle I (a) and cycle II (b).

To follow the changes of the molecules and the nano-assemblies, the samples were monitored by UV–vis spectroscopy, as shown in Figure 3. The non-irradiated sample shows a broad band at λ = 550 nm, which corresponds to Flavy as evident in comparison with Figure 1. The difference compared to the pure Flavy spectrum can be understood with the presence of both forms ***A*** and ***AH^+^***. The band of the photoacid is overlaid by the Flavy spectra, which causes the bands at λ = 300 nm to appear quite small. In cycle I the irradiation of the photoacid leads to a decrease of the band at λ = 550 nm, while at the same time a peak emerges at λ = 300 nm. In addition, the band corresponding to the photoacid becomes less distinct. These two phenomena indicate the deprotonation of the photoacid and the transformation of Flavy from the ***A*** to the ***B*** form. After keeping the assemblies in the dark overnight the peak at λ = 550 nm completely disappeared, while at the same time the intensity of the band at λ = 300 nm increased. These spectral changes correspond to the transformation of the remaining ***AH^+^*** to the ***B*** form of Flavy, which is also due to the decrease of the pH value from pH 7.0 to pH 4.8 after irradiation of the photoacid. The addition of HCl then leads to significant changes in the UV–vis spectra: The band of the ***AH^+^*** form of Flavy appears and the band at λ = 300 nm decreases significantly. The bands of the photoacid become as distinctive as in the beginning indicating that the photoacid is present in its protonated state. For cycle II, the changes upon the addition of HCl are the same as for the Flavy only solution, suggesting the presence of ***AH^+^***. At a pH value of 5.0 the intensity of the band at λ = 550 nm decreases (blue curve) meaning that ***AH^+^*** transforms to form ***B*** from Flavy leading to state **D**. This can also be seen at the band at λ = 300, which increases again in intensity. The irradiation “of the photoacid“ with λ = 300 nm causes a decrease of the band at λ = 300 nm (magenta curve). At the same time a band appears at λ = 400 nm corresponding to ***Cc***. Also, the bands corresponding to the photoacid become less distinct upon irradiation. After keeping the solution in the dark and changing to an acidic environment the band at λ = 550 nm reappears (orange curve), while at the same time the bands of the photoacid again become more distinct. Overall, according to the UV–vis spectra the cycles I and II are reversible. Quantitatively, comparing the absorbance of Flavy before and after completion of the different cycles shows that close to 75% of Flavy revert back (data given in Table 1). Likely the reason for the incomplete reversibility is a photoprotective effect which hinders the open form ***Cc*** to fully revert back when bound in the assemblies.

**Table 1:** Percentage of the absorption of the Flavy molecule at λ = 500 nm after a complete cycle.

	cycle I	cycle II

Δ*A* (Flavy)	0.114	0.125
*A* (Flavy)	75%	72%

**Figure 4:**
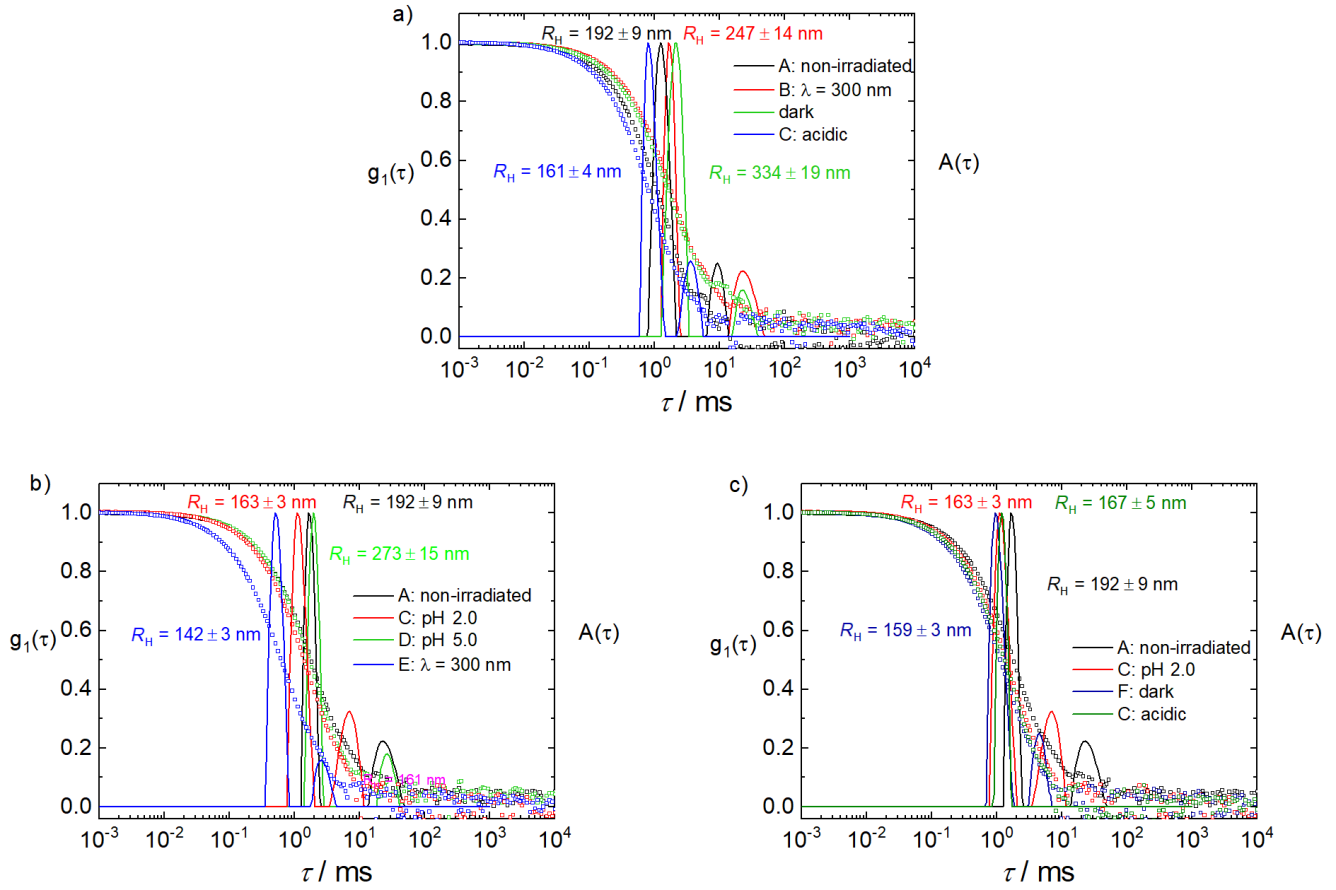
Dynamic light scattering: Electric field autocorrelation function g_1_(τ) and distribution of relaxation times A(τ) at a scattering angle of θ = 90° for cycle I (a) and cycle II (b,c). Cycle II is separated for better distinguishability: b) the states **A**, **C**, **D**, **E** and c) the states of **A**, **C**, **F**, **C**.

To investigate how these differences in molecular building block structure affect the assembly nanoscale size and structure, dynamic and static light scattering (DLS, SLS) were performed. DLS measures the intensity fluctuations caused by the diffusion of the particles, which is quantified in the intensity and electric field autocorrelation functions. The electric flied autocorrelation functions g_1_(τ) are transferred into the distributions of relaxation times A(τ) (both shown in Figure 4), corresponding to the size distributions of the particles. Figure 4 shows the data at a scattering angle of θ = 90° for cycle I and II to indicate these size distributions of the assemblies formed in solution. For a quantitative analysis and the hydrodynamic radii quoted, angular extrapolated values are used. For each sample of both cycles, one main peak is visible, that is, one predominant assembly size. The size differs depending on the cycle and cycle step, lying between a hydrodynamic radius of *R*_H_ = 142 nm and *R*_H_ = 332 nm; that is, significant size changes occur upon triggering. A second smaller peak that in some samples occurs at higher relaxation times corresponds to a few larger particles. Here it should be noted that the displayed distributions represent intensity-weighted distributions and in terms of a number distributions these larger species in most cases are almost neglectable. This suggests that overall assemblies with one preferred size form. The hydrodynamic radii are given in Table 2 and will be considered in the following.

**Table 2:** Size of the assemblies at *l* = 0.9 and *k* = 1.0 at various steps of the two cycles.

*R*_H_ [nm]	non-irrad.	pH 2.0	pH 5.0	irradiated	dark	acidic

cycle I	192	–	–	247	334	161
cycle II	192	163	273	142	159	167

**Figure 5:**
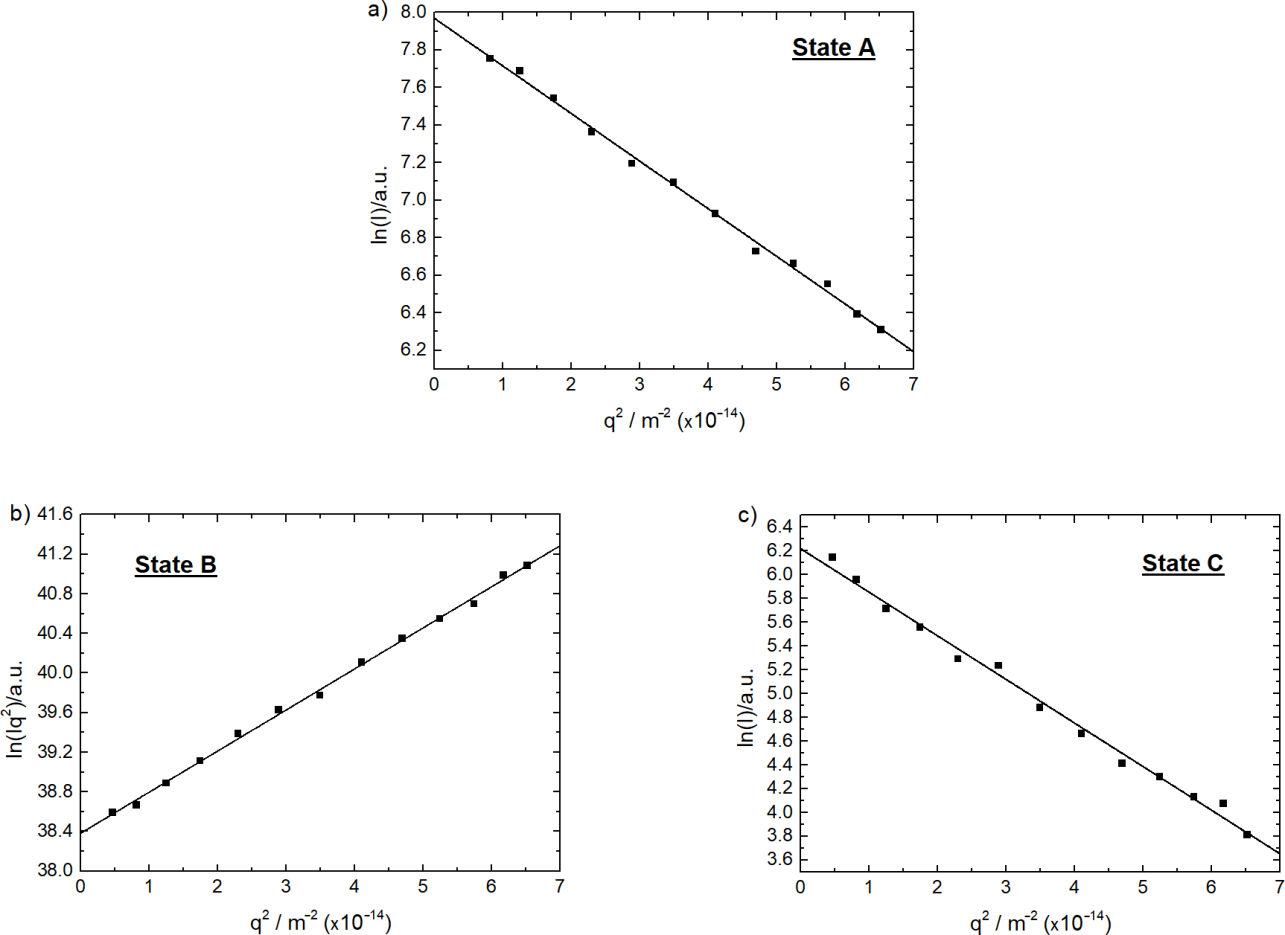
Static light scattering data from the assemblies of cycle I; a) **A**, non-irradiated, spherical particles; b) **B**, irradiated, disc-like particles; c) **C**, dark and acidic environment, spherical particles.

After mixing the building blocks, DLS reveals a size of *R*_H_ = 192 nm, while the SLS indicates the structure of a sphere (linearity in a Guinier plot, Figure 5a). In cycle I, the excitation of the photoacid to state **B** leads to an increase in size to *R*_H_ = 247 nm. At the same time, the assembly structure changes to a disc-like (Figure 5b, linearity in a thickness-Gunier plot of ln(Iq^2^) versus q^2^, where Iq^2^ represents the thickness scattering function [24,36]). Keeping the solution in the dark and after addition of HCl to achieve state **C** the size of the assemblies decreases to *R*_H_ = 161 nm. At the same time, the structure changes back to a sphere (Figure 5c). To make a statement on the reversibility, it is necessary to compare this state of cycle I with the second state of cycle II. Cycle II shows a decrease in size from *R*_H_ = 192 nm (state **A**) to *R*_H_ = 163 nm (state **C**). The size of *R*_H_ = 163 nm for state **C** of cycle II is the same size as at the end of cycle I. This leads to the conclusion that cycle I is reversible. This further is supported with the same sphere-like structure. The next step in cycle II is the hydrolysis of the hydroxyflavylium molecule to state **D**, which causes an increase in the size to *R*_H_ = 273 nm. The subsequent irradiation of the assemblies does not only decrease the size to *R*_H_ = 142 nm but also changes the structure to a disc (Figure S2 in Supporting Information File 1). Combined with the results of cycle I this suggests that the changes of the structure depend on the deprotonation of the photoacid due to irradiation and the subsequently higher charged molecule. The last two steps for cycle II are as in cycle I and the size and structure of the assemblies switch back to the starting point. Interestingly, these two cycles differ regarding the behavior after the irradiation of the photoacid. This is due to the different states of Flavy being present after the irradiation. In case of cycle I the hydroxyflavylium molecule is in form ***B*** according to the UV–vis data, while for cycle II Flavy is in the form of ***Cc***. Thus, ternary assemblies of a certain size are formed and the size and shape can be addressed with different triggers including irradiation and pH changes in different switching cycles. The reasons for the resulting sizes will be discussed further below.

**Figure 6:**
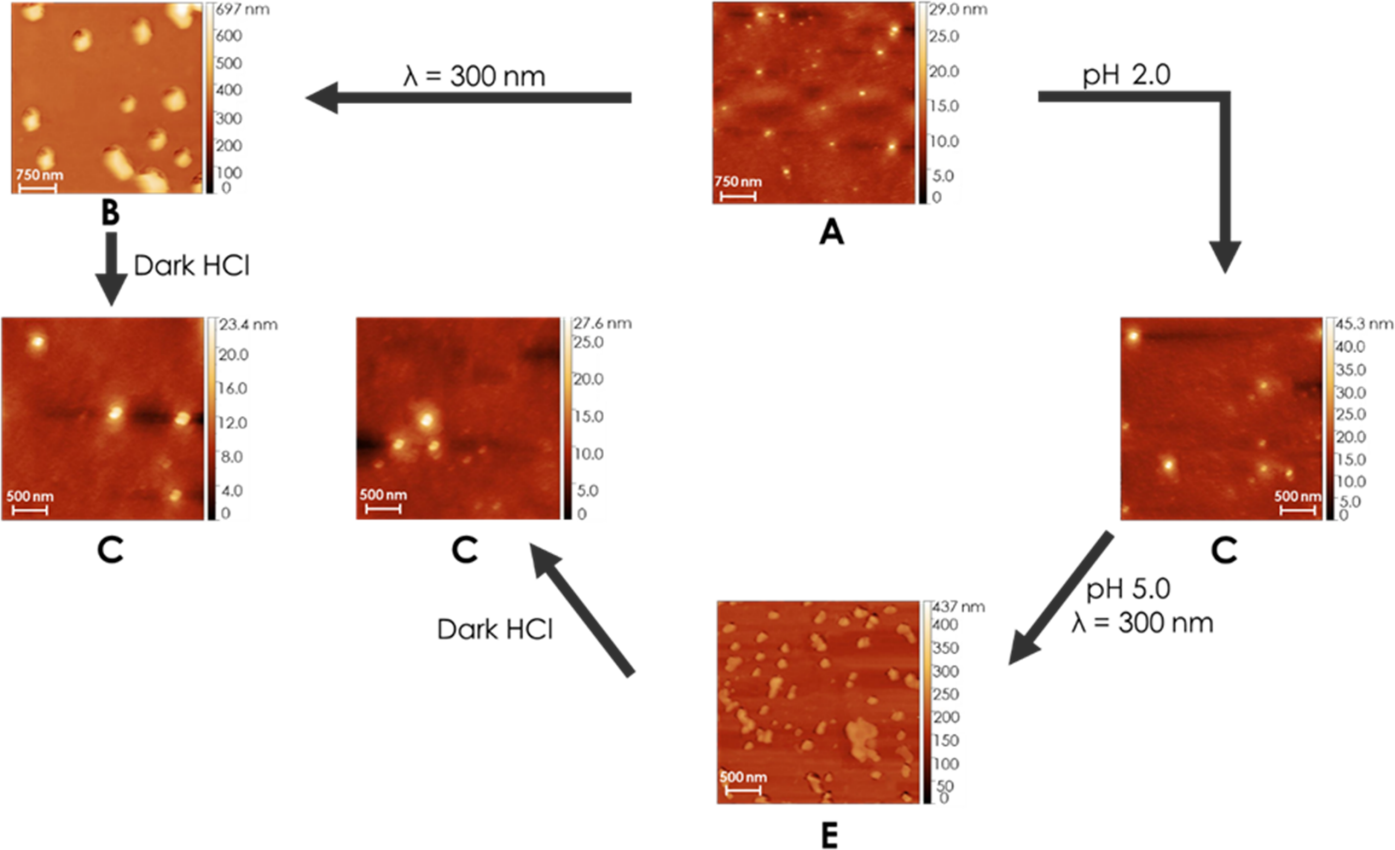
Comparison of cycle I and cycle II in AFM.

To further investigate the structure, the samples were also studied by AFM. For this, assembly solutions where drop cast on mica surfaces. The results are given in Figure 6 and overall they are in agreement with the DLS results. To compare the sizes of the assemblies quantitatively, the volumes of the nanoparticles measured by AFM on the surfaces are calculated, converted into a hypothetical radius of a volume-equivalent spherical particle, and compared to the hydrodynamic radii from DLS. The average radii calculated from the AFM volumes are given in Table 3 and Table 4. As can be seen the relative size changes are very similar to the ones observed in DLS and again show the reversibility of both cycles. As reported for other systems, the structures in AFM are substantially smaller than in DLS due to the shrinking upon drying in the AFM sample preparation [40,60,83–85].

**Table 3:** Radii of the structures in cycle I obtained from AFM volume considerations.

cycle I	*R*_AFM_ for sphere [nm]	*R*_DLS_ [nm]

**A**	155	192
**B**	205	247
**C**	63	161

**Table 4:** Radii of the structures in cycle II obtained from AFM volume considerations.

cycle II	*R*_AFM_ for sphere [nm]	*R*_DLS_ [nm]

**A**	155	192
**C**	83	163
**D**	231	273
**E**	99	142
**C**	84	167

**Figure 7:**
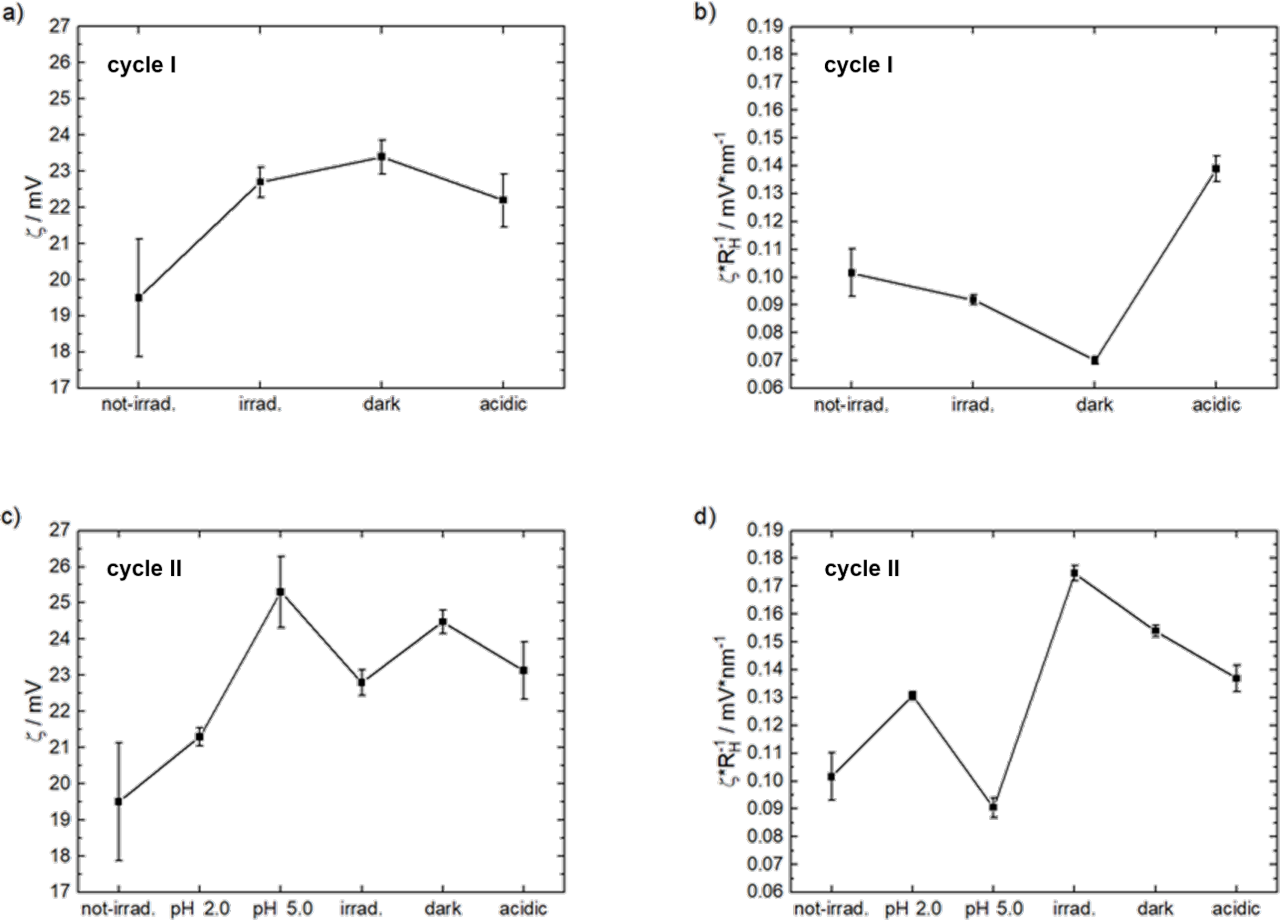
a) ζ-Potential and b) effective surface charge density for cycle I; c) ζ-potential and d) effective surface charge density for cycle II.

Further, the ζ-potential was determined to understand the changes in the charge-related properties and triggered changes of the assemblies and the results are given in Figure 7. All values are positive, showing the overall cationic charge of the assemblies. The excess charge is what stabilizes the few-hundred-nanometer assemblies with a certain size in solution. Figure 7 also displays the effective surface charge density as a possible determining parameter: As the ζ-potential represents a charge per radius (all as effective values at the shear plane of the particle diffusing in solution), the magnitude of the effective surface charge density is obtained by dividing the ζ-potential by *R*_H_. Previously we have reported that the effective surface charge density significantly controls the supramolecular particle size in electrostatic self-assembly [4,86–87]. (The effective surface charge density considers the effect of all particle charges at the shear plane.)

For cycle I, the ζ-potential first increases, while the effective surface charge density decreases upon irradiation. Due to the photoacid changing from a di- to a trianionic molecule under irradiation and likely also due to the transformation of the Flavy molecule from the ***A*** to the ***B*** form, which leads to the addition of a fifth hydroxy group, more binding sites become available for the association with the poly(allylamine), leading to an increase in assembly size: More multivalent small molecules bind to the polyelectrolyte, interconnecting more polyelectrolyte molecules into larger aggregates. At the same time, the effective surface charge density decreases due to the reduced number of excess positive charges in the assembly. In the next step of cycle I, the assemblies are kept in the dark. This leads to the further transformation of Flavy from ***A*** to ***B***, as was observed by UV–vis spectroscopy. In this case the effective surface charge density further decreases and the size of the particles further increases. The addition of HCl then leads to state **C** causing a decrease in the ζ-potential and an increase in the effective surface charge density. This is due to the transformation of the Flavy molecule from the ***B*** form to ***AH^+^***, which creates a cationic charge on the Flavy molecule. A decreased electrostatic driving force due to the repulsion of the positive charges of Flavy and the polyelectrolyte lead to the decrease in particle size. In other words, again the increased effective surface charge density allows to stabilize the assemblies with a smaller size.

Figure 7 also displays the ζ-potential and the effective surface charge density for cycle II. Changing the pH value from pH 7.0 to pH 2.0 leads to the transformation to the ***AH^+^*** form of the **C** state with a new cationic charge. This results in a slight increase in both the ζ-potential and the effective surface charge density. Also, the value is again similar to the last step of cycle I, once more indicating a reversible scenario. Upon the addition of NaOH to change the pH value to pH 5.0, the effective surface charge density decreases. The transformation to the hydrolyzed form of the hydroxyflavylium molecule at state **D** leads to the removal of the positive charge and the addition of an extra binding site. Due to that, the ζ-potential increases while at the same time the effective surface charge density decreases followed by a destabilization of the smaller assemblies and an increase in size. The subsequent irradiation at state **E** of the assemblies again leads to the formation of the more highly charged photoacid and the ***Cc*** form of Flavy, accompanied by an increase in the effective surface charge density corresponding to a decrease in size from *R*_H_ = 273 nm to *R*_H_ =142 nm. This is followed by a decrease of the ζ-potential. Upon keeping the assemblies in the dark and adding HCl the assemblies transform back to the original assemblies in the acidic environment at state **C**, demonstrating the reversible and dynamic character of the process.

### Thermodynamic analysis of the assembly process

**Figure 8:**
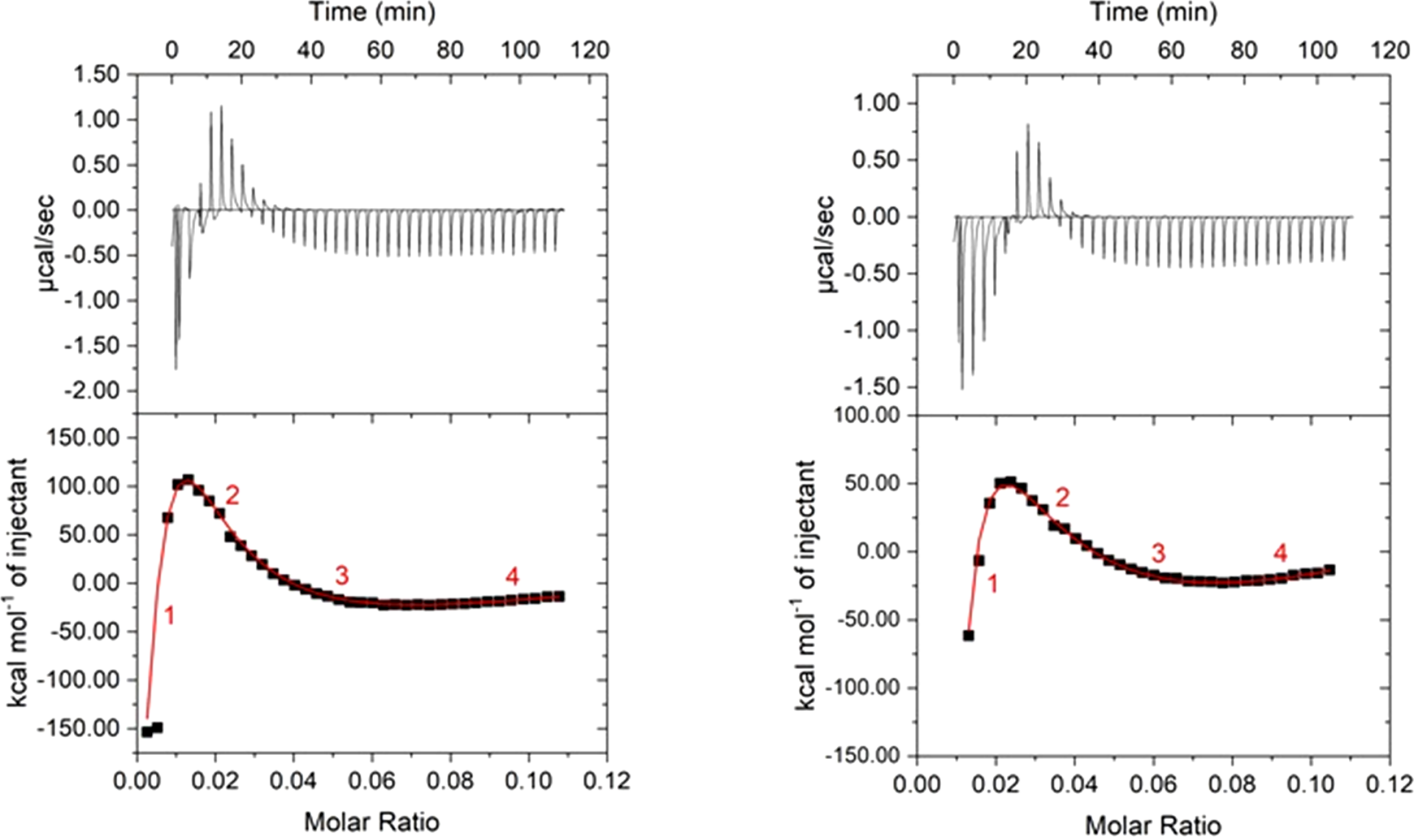
Isothermal titration calorimetry of poly(allylamine) into the cell containing Flavy and 1N36S in aqueous solution. Top: Raw data of the titration, bottom: titration curve and fit according to four binding sites; left: at pH 7.0; right: at pH 5.0. The molar ratio on the *x*-axis represents the addition of poly(allylamine).

For understanding the differences of the assemblies, isothermal titration calorimetry (ITC) was performed to elucidate the thermodynamics and to gain insight into the building block interactions in the assembly process. Basically, ITC measures the association heat (enthalpy) and as it is performed as a full titration, the stoichiometric information and further thermodynamic parameters can be deduced. Figure 8 and Table 5 show the raw data and the analysis of the titration of the polyelectrolyte into a photoacid/hydroxyflavylium solution at pH 5.0 and pH 7.0. The change of the pH value from pH 7.0 to pH 5.0 leads to an addition of one hydroxy group. This means that the number of possible hydrogen bonds increases from four at pH 7.0 to five at pH 5.0. Yet, the added hydroxy group is in a position where steric hindrance can play a major role. Furthermore, Flavy and the photoacid also form nano-assemblies on their own, which are also dependent on the pH value (see Supporting Information File 1). The assemblies are expected to form due to hydrogen bonds and ionic-dipole interaction. Already the raw data (Figure 8) clearly show an exothermic, an endothermic, and another exothermic region, that are at least three binding steps and thus an overlay of different binding types. The data for both pH values can be fitted with the so-called model of “sequential binding” with four binding steps (three binding steps do not describe the data adequately). This model considers the interaction at multiple binding sites, which might be identical or non-identical. Thus, the model is generally applicable for any possible scenario with more than one ligand. In addition to the enthalpy, the titration curve contains direct information of the stoichiometries and equilibrium binding constants *K*_x_, which are related to the free energy change Δ*G* and thus due to Δ*G* = Δ*H* − *T*Δ*S*, also the entropy change Δ*S* is revealed. Table 5 summarizes the results from the full titration curve fitting. The values correlate to the binding of one monomer of the polymer to Flavy and/or photoacid.

**Table 5:** ITC results at pH 7.0 and pH 5.0 from the sequential binding fit. The values correspond to the binding of one monomer of the polymer with Flavy and/or photoacid.

	pH 7.0	pH 5.0

*K*_1_ [M^−1^]	5.81 × 10^4^	7.30 × 10^5^
Δ*H*_1_ [kJ/mol]	−4.3	−106.1
Δ*S*_1_ [kJ/mol/K]	−0.01	−0.36
Δ*G*_1_ [kJ/mol]	−0.10	−0.13
*K*_2_ [M^−1^]	3.6 × 10^4^	4.94 × 10^5^
Δ*H*_2_ [kJ/mol]	64.5	46.9
Δ*S*_2_ [kJ/mol/K]	0.22	0.16
Δ*G*_2_ [kJ/mol]	−0.1	−0.12
*K*_3_ [M^−1^]	2.41 × 10^6^	7.15 × 10^5^
Δ*H*_3_ [kJ/mol]	−172.7	−223.6
Δ*S*_3_ [kJ/mol/K]	−0.56	−0.75
Δ*G*_3_ [kJ/mol]	−0.58	−0.13
*K*_4_ [M^−1^]	3.02 × 10^6^	6.59 × 10^5^
Δ*H*_4_ [kJ/mol]	75.5	84.1
Δ*S*_4_ [kJ/mol/K]	0.25	0.28
Δ*G*_4_ [kJ/mol]	−0.14	−0.13
Δ*H*_total_ [kJ/mol]	−37.0	−198.7
Δ*S*_total_ [kJ/mol/K]	−0.1	−0.44
Δ*G*_total_ [kJ/mol]	−0.92	−0.51

For pH 7 (Figure 8), the data reveal that the first and third binding site are exothermic with Δ*H*_1_ = −4.3 kJ/mol and Δ*H*_3_ = −172.7 kJ/mol per polyelectrolyte binding site, respectively, which shows that strong ionic interactions and hydrogen bonding constitute these binding processes. Probably these values are a combination of the hydrogen bonding of Flavy with poly(allylamine) and the electrostatic interaction of the photoacid with the cationic polyelectrolyte. Generally, typical hydrogen-bonding strengths range from −10 kJ/mol to −30 kJ/mol in aqueous solutions [88–89]. A hydrogen bonding of quaternary amines with hydroxy groups is not common but has been observed [90–92]. The electrostatic interaction of naphthol-based molecule ions are known to be at least of Δ*H* = −50 kJ/mol [32]. This suggests that here not only one charge interacts, instead, for Δ*H*_3_ it has to be between three charges and one charge plus four hydrogen bonds, which corresponds to one Flavy for example. In difference, the second and fourth binding step are endothermic with Δ*H*_2_ = 64.5 kJ/mol and Δ*H*_4_ = 75.5 kJ/mol. Here, the association is entropically driven with Δ*S*_2_ = 0.22 kJ/mol/K and Δ*S*_4_ = 0.22 kJ/mol/K, indicating that an association occurs because of hydrophobic effects, likely due to the slight hydrophobic nature of Flavy. The total free energy Δ*G*_total_ is negative, which shows that the overall assembly process is spontaneous. The molar ratio (*x*-axis in Figure 8) representing the increasing addition of poly(allylamine) in the experiment can be translated into the previously introduced loading ratio *l* (Equation 1), which decreases from left to right (9.6 ≥ *x* ≥ 0.14). The first thermodynamic interaction process can be followed down to a loading ratio of *l* = 0.98 (up to a molar ratio of 0.01) for pH 7.0. In this region there is an excess of negative charges present, in accordance with the exothermic nature of the interaction. The small magnitude of Δ*H*_1_ originates from the assemblies of Flavy and the photoacid being present in the cell. These molecules already interact before the experiment starts and need to (partly) disassemble in the formation of the assemblies with the polymer such that poly(allylamine) competes with the Flavy–photoacid interaction. The rearrangement that takes place then also shows that the interaction of the poly(allylamine) with Flavy and the photoacid is preferred. The competition scenario can also be elucidated when comparing the Δ*H*_1_ value to the data from a poly(allylamine)–Flavy and a poly(allylamine)–photoacid ITC experiment that we have performed for comparison. In both binary cases Δ*H*_1_ exceeds the value in the ternary system, which again shows that poly(allylamine) competes with the Flavy–photoacid interaction in the ternary experiment. The second step is present from *l* = 0.98 down to a loading ratio of *l* = 0.33 (up to a molar ratio of 0.035). The endothermic nature and the entropically favored binding indicate the occurrence of hydrophobic interactions, while also a small counterion release may contribute (likely, it is ionic repulsion due to the excess of positive charges (*l* < 1.0) in combination with changes in hydration shells that make this region endotherm). The last binding step starts at *l* = 0.16 (molar ratio 0.075), equal to 17 monomer units per poly(allylamine) to one Flavy and photoacid molecule each, corresponding to 15 Flavy and photoacid molecules each per one polyelectrolyte chain. The highly exothermic process indicates that up to this loading ratio the attractive electrostatic interaction contributes to the association again. This can be understood with the fact that likely not every anionic charge can interact with a cationic charge due to steric reasons. No more ionically driven association occurs at even lower loading ratios for *l* < 0.16 as there are too many cations in the assemblies. The results for the assemblies at pH 5.0 are similar. The main difference is the first binding step ranging down to *l* = 0.5 (up to a molar ratio of 0.022) for pH 5.0, that is, farer than for pH 7.0. At pH 5.0 Flavy exists in the ***B*** form, which has one more hydroxy group. Thus, more binding groups that may attach to the polyelectrolyte are available and thereby the first exothermic region extends until more poly(allylamine) has been added. Along the same line, the first binding enthalpy is significantly lower at pH 7.0, which can likely be attributed to a difference in the Flavy–photoacid assemblies at the two pH values.

**Figure 9:**
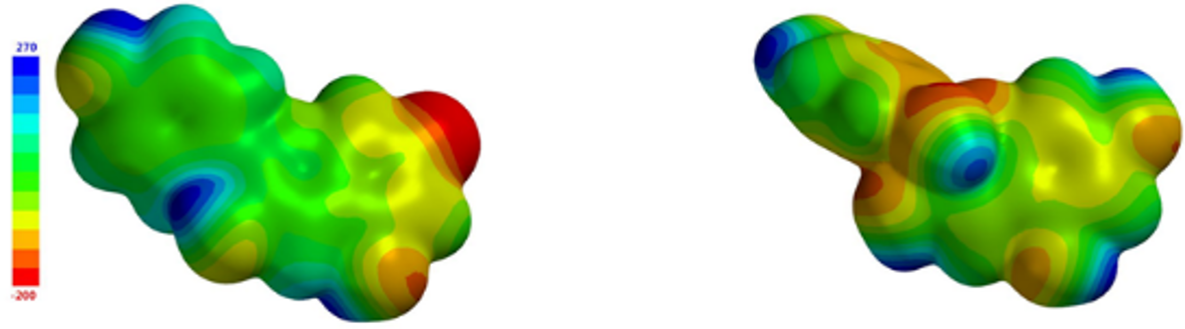
Polar surface area of Flavy in form of ***A*** (left) and ***B*** (right).

The difference in the interaction strength may be understood based on the molecular parameters of the building blocks and in particular the polar surface area (PSA), which relates to the three-dimensional charge distribution of the molecule. The PSA results from the area of the polar groups in the molecule taking into account the substituents. Previously we have shown for a simpler model system that the PSA correlates with the interaction of the molecules, in particular, the dye–dye electrostatic repulsion [31]. Hence, the lower the PSA the better the dye molecules can interact with each other through π–π interactions. Comparing the results of Flavy in Figure 9 at pH 5.0 and at pH 7.0 shows that the Flavy molecules should interact more strongly with themselves and with the photoacid at pH 7.0, causing a lower enthalpy for the first binding step at pH 7.0 as compared to pH 5.0. The strong pH effect also becomes evident in the differences in the total enthalpy Δ*H*_total_ and free energy Δ*G*_total_. These also reflect in the final size of the assemblies at *l* = 0.9: At pH 5.0 the size of the assemblies is *R*_H_ = 516 nm, at pH 7.0 the size is only *R*_H_ = 159 nm. While the total enthalpy Δ*H*_total_ is significantly more negative for pH 5.0 than for pH 7.0, the free enthalpy is somewhat more negative for pH 7.0 due to the additional strong entropic effect in the whole scenario. This is different to the previously detected Δ*G*-encoded particle size in the electrostatic self-assembly [37], and demonstrates the higher complexity of the system under investigation here. In conclusion the differences between the two pH values originate in the structure and the PSA of the two molecules and can be related to the sizes and the dependency of the loading ratio observed in DLS.

### Effect of loading ratio and concentration ratio

The samples discussed above were prepared at *l =* 0.9 and *k* = 1.0. For the binary polyelectrolyte–dye assemblies studied previously it was found that the overall loading ratio significantly determines the properties of the nano-assemblies [4,34,40,86]. In addition, due to the combination of two switchable molecules with the polymer it is also important to consider the effect of different ratios of hydroxyflavylium to photoacid. In the following, first the loading ratio is varied and secondly, the loading ratio is kept constant and the influence of the concentration ratio on the nano-assemblies is studied.

**Figure 10:**
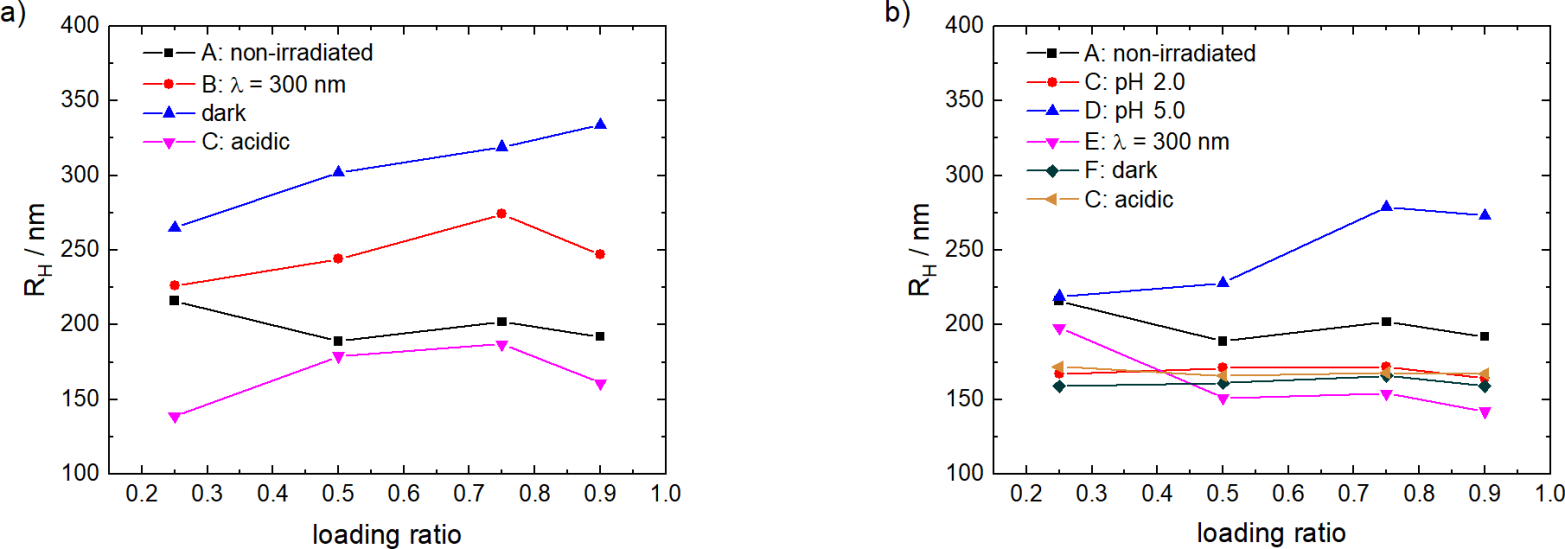
Hydrodynamic radii of the nano-assemblies as function of the loading ratio: a) cycle I, b) cycle II.

The ITC measurements above already have revealed that the thermodynamics depended on the loading ratio. To elucidate how it influences the size of the particles, DLS was measured at the different binding steps. Figure 10 shows the DLS results in dependence on the loading ratio below *l* = 1.0. The structures with a loading ratio of *l* = 0.125 are polydisperse with a particle size of *R*_H_ = 17 nm to *R*_H_ ≈ 1000 nm. This is probably due to the excess of polyelectrolyte leading to the formation of loose and broadly distributed aggregates. Before irradiation, at a loading ratio of *l* = 0.25 the assemblies are larger than for *l* = 0.5, *l* = 0.75, and *l* = 0.9. This difference corresponds to the ITC. The loading ratios 0.5 ≤ *l* ≤ 0.9 lie in the range of the second binding step that is driven by the hydrophobic effect, whereas the loading ratio *l* = 0.25 is in the range of the third binding step where enthalpic interactions dominate again. Cycle II shows similarity to cycle I. For both cycles, the largest size range that is covered when going through all switching steps – that is from *R*_H_ ≈ 150 nm to *R*_H_ ≈ 350 nm for cycle I and to *R*_H_ ≈ 300 nm for cycle II – is realized for the loading ratio *l* = 0.9, which represents the model case discussed in detail above.

**Figure 11:**
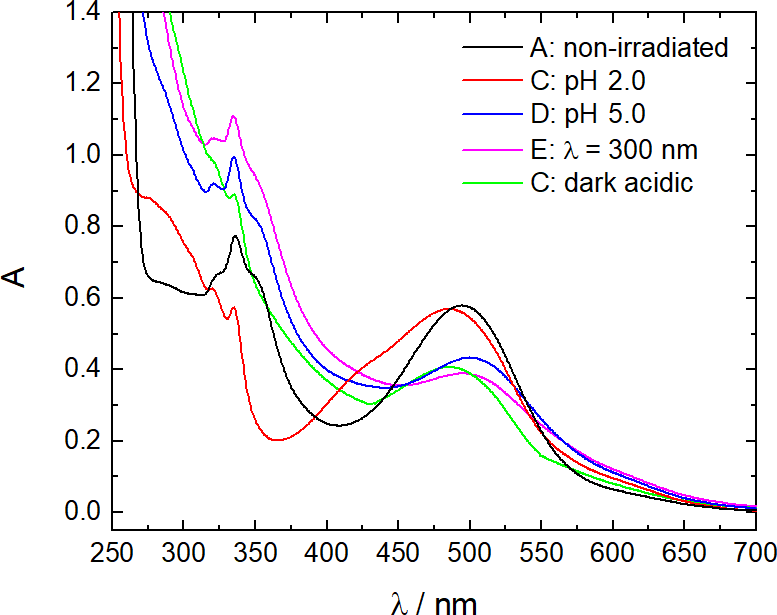
UV–vis spectra of the nano-assemblies of cycle II at *l* = 0.75.

Additionally, UV–vis spectra were recorded to check the reversibility of the systems with varying loading ratios. Therefore, the percentages of Flavy turned back at the end of the cycles at state **C** are calculated, as given in Table 6. The spectra for *l* = 0.9 and *l* = 0.75 are depicted in Figure 11. The highest percentage of reformed Flavy is found for *l* = 0.9, while the reversibility is less expressed with decreasing loading ratios for both cycles. Since it is independent on the cycle and dependent on the loading ratio the origin likely lies in the fact that with a decreasing loading ratio more building blocks protect the Flavy molecule from transforming back by intermolecular interactions.

**Table 6:** Percentages of the absorption of the Flavy molecule at the **C** states at λ = 500 nm after a complete cycle depending on the loading ratio.

	*l* = 0.25	*l* = 0.5	*l* = 0.75	*l* = 0.9

cycle I	56%	58%	66%	75%
cycle II	47%	56%	67%	72%

Further, the concentration ratio of the photoacid to Flavy may also have a strong influence on the structures and properties of the assemblies. To analyze this effect, the loading ratio is kept constant at *l* = 0.9 and the concentration of Flavy at *c*(Flavy) = 1 × 10^−4^ mol/L, while the concentration of the photoacid is varied. The DLS results collected in Table 7 show that at an excess of the photoacid in both cycle I and cycle II lead to the formation of polydisperse structures in the first few steps except for the acidic environment in cycle II. Furthermore, the assemblies from both cycles start to precipitate after irradiation of the photoacid. A reason for that is the higher concentration of the photoacid, which leads to more interconnections after the irradiation. An excess of the Flavy molecules was not measured, since it is necessary to have sufficient photoacid molecules present to transform the Flavy upon the irradiation of the photoacid.

**Table 7:** Concentration ratio dependence: hydrodynamic radii for the different states of both cycles. The samples of *k* > 2 started to precipitate (prec.) after irradiation.

*R*_H_ [nm]		non-irrad.	pH 2.0	pH 5.0	irrad.	acidic

*k* = 1.0	cycle I	192			247	161
	cycle II	192	163	273	142	167
*k* = 2.0	cycle I	260 + 630			prec.	prec.
	cycle II	260 + 630	13 + 118	61	prec.	prec.
*k* = 4.0	cycle I	18 + 211			prec.	prec.
	cycle II	18 + 211	13 + 138	174	prec.	prec.

**Figure 12:**
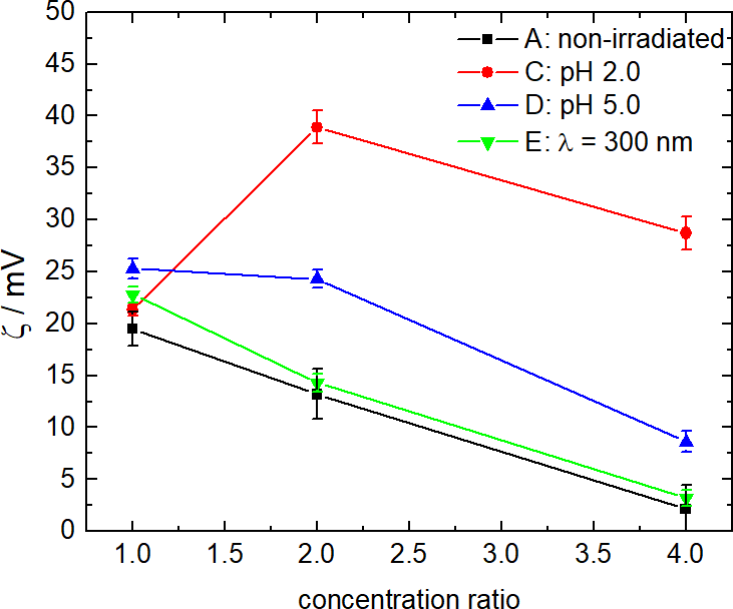
ζ-Potential of the nano-assemblies of cycle II depending on the concentration ratio.

The changes measured in DLS again can also be followed by the ζ-potential (Figure 12). Prior to irradiation, the ζ-potential decreases with an increasing concentration ratio. Due to that the assemblies are less stable. This changes for *k* ≥ 2 at pH 2.0, where the ζ-potential significantly increases. Since there are at least twice as many photoacid molecules than Flavy molecules, likely, the ***AH^+^***-cationic poly(allylamine) repulsive contribution is not as determining as for *k* = 1.0 and due to that more cationic molecules can be built into the assembly. This is in accordance with the more defined assemblies formed for *k* = 1.0 as compared to the polydisperse samples for 4.0 ≥ *k* ≥ 2.0.

Thus, both the charge and concentration ratios have a strong influence on the characteristics of the assemblies. At an excess of negative charges, the particles precipitate due to the interconnection with the trianionic photoacid. The structural changes at a loading ratio *l* < 1.0 can be understood in conjunction with the ITC measurements. At an excess of the photoacid, the assemblies are unstable and start to precipitate after irradiation, likewise for a total excess of negative charges as expressed in the loading ratio.

### Influence of the mixing order on the nano-assemblies

**Scheme 5:**
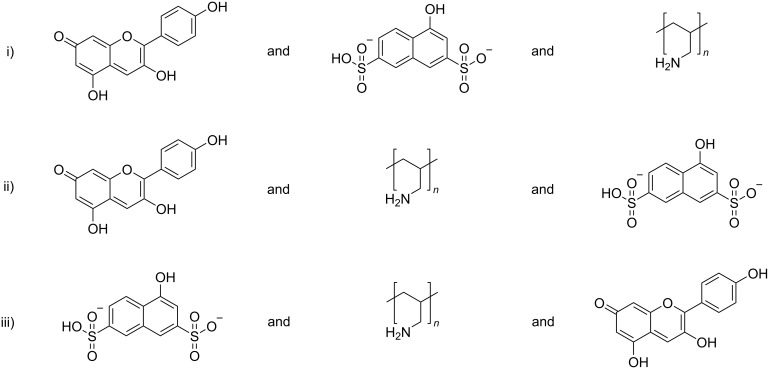
Different mixing orders of the assemblies*.* The major part of this study focuses on route i.

The samples discussed above were prepared by first mixing hydroxyflavylium and photoacid stock solutions, and then adding the polyelectrolyte (route i). To study a possible influence of the preparation route, the building blocks were mixed in different order, while the concentration and loading ratios were the same. An overview is given in Scheme 5.

Applying route ii, where both cationic building blocks Flavy and poly(allylamine) are mixed first, the aggregation yields clearly polydisperse ternary samples (Table 8 and Table 9). For understanding, it is of interest to consider the intermediate bi-component system, which already leads to polydisperse samples for loading ratios *l* < 1.0, as measured by DLS (results not shown). The following addition of the photoacid cannot change the polydispersity, i.e., in this case the system is kinetically trapped. In contrast, mixing the photoacid with the polyelectrolyte first (route iii) leads to similar assemblies as in route i. Not only are the sizes similar but also their response is the same. The same applies for cycle I and cycle II. The fact that the same sizes result via different pathways (route i and route iii), furthermore supported by the reversibility in the cycles in route i as discussed in the paragraphs above, strongly supports that these assemblies are thermodynamically controlled. The structures only depend on the state independent of the preparation pathway.

**Table 8:** Size of the assemblies at different mixing orders of cycle I.

*R*_H_ [nm]	route i	route ii	route iii

non-irrad.	192	polydisperse	199
irrad.	247	polydisperse	262
dark	334	polydisperse	348
acidic	161	polydisperse	146

**Table 9:** Size of the assemblies at different mixing orders of cycle II.

*R*_H_ [nm]	route i	route ii	route iii

non-irrad.	192	polydisperse	200
pH 2.0	163	polydisperse	181
pH 5.0	273	159	319
irrad.	142	polydisperse	158
dark	159	polydisperse	214
acidic	167	polydisperse	174

## Conclusion

In conclusion we have developed a novel reversible multi-switchable system consisting of a cationic polyelectrolyte, a hydroxyflavylium molecule (Flavy), and a photoacid. Ternary assemblies with sizes in the hundred-to-few hundred nanometers range in aqueous solution exhibit a multi-addressable size and shape. The concept exploits the unique property of the photoacid to form a more highly charged molecule and to switch the Flavy molecule in the same step when excited by light irradiation. Due to the network of possible reactions of Flavy, self-assembly can be accessed and triggered in a number of ways. While this study focused on the first proof of concept and the relation of molecular and nanoscale switching, a deeper understanding of the molecular binding effects may be considered in future studies. The type of the photoacid-based assembly presented here bears potential, for example, for delivery where the assembly property changes may provide a desirable transformable platform for tunable and smart transport.

## Experimental

The poly(allylamine) with *M* = 15000 g/mol, the photoacid 1-naphthol-3,6-disulfonate (>90%), and pelargonidin chloride were purchased from Sigma-Aldrich. Deionized water was filtered through two 25 mm syringe filters with a hydrophile membrane consisting of polytetrafluoroethylene with 200 nm pore size.

Prior to sample preparation, a stock solution of each chemical was prepared in deionized water with Millex-LG water at pH 7.0. The pH value was detected with a HI 221 Microprocessor pH meter and adjusted by adding NaOH or HCl (1 N, filtered with Millex-LG filter). For the photoacid and the anthocyanidin, the stock solutions were stored under light exclusion. After the addition of Flavy to water the photoacid is added to the solution under stirring and light exclusion. After two minutes the poly(allylamine) is added. The final concentration of *c*(Flavy) = 1 × 10^−4^ mol/L was kept the same for all samples. The concentration of the polyelectrolyte and the photoacid were varied according to the anticipated concentration and loading ratios.

For UV light irradiation, a UV lamp UVLM-28 EL from analytikjena with 8 W was used. Dynamic light scattering was carried out at an ALV 5000 correlator with 320 channels (ALV GmbH, Langen, Germany), an ALV CGS 3 goniometer, and a red HeNe laser (λ = 632.8 nm, 20 mW). The samples were measured over a scattering angular range of 30° ≤ θ ≤ 150° in steps of 10° for a duration of 50 seconds. Via the Siegert relation the intensity autocorrelation function g^2^(τ) was transferred into the electric field autocorrelation function g^1^(τ). By a regularized inverse Laplace transformation, the electric field autocorrelation function g^1^(τ) was transformed into the distribution of relaxation times A(τ). The apparent diffusion coefficients *D*_app_ were calculated by Equation 3

[3] $$D_{\text{app}}=\frac{\Gamma }{{}^{q^{2}}}$$

The diffusion coefficients *D*_0_ were obtained via extrapolation to zero scattering vector square and via Stokes–Einstein relation the hydrodynamic radii *R*_H_ resulted. With the same setup static light scattering was measured. In static light scattering, the average sample, solvent (water), and standard (toluene) scattering intensities were recorded in dependence on the scattering angle. The absorption spectra were recorded on a SHIMADZU UV Spectrophotometer (UV-1800) with a slit width of 1.0 nm and a range of 200 nm ≤ λ ≤ 800 nm. The spectra were recorded against air as a reference. For all measurements 10 mm quartz cuvettes were used. Atomic force microscopy (AFM) was performed using a NanoWizard 4 from JPK instrument (Berlin, Germany) operated in the tapping-mode with a fixed-spring cantilever holder and a USC-F0.3-k0.3-10 ultrashort cantilever with a force constant of 0.3 Nm^−1^. The cantilever has been tested and established for organic samples of low height [67,84]. The AFM samples were prepared by drop-casting the solution on cleaved mica substrate. The samples before and after irradiation were blow-dried for 15 min. The images were analyzed using Gwyddion 2.47 and carefully examined for artifacts [93–94]. The volumes of the AFM structures have been calculated using a spherical cap as model structure. ζ-Potential measurements were carried out with a Zetasizer Nano ZS analyzer with a 4 mW HeNe laser (λ = 633 nm; Malvern Instruments Ltd., U.K.). The solutions were placed in folded capillary cells (DTS 1070). After applying an electric field across the sample solution, the electrophoretic mobility was measured by using the technique of laser Doppler anemometry. By using the Smoluchowski approximation, the ζ-potential is calculated from the electrophoretic mobility. The measurements were performed at 20 °C and repeated three times to gain an average value. Isothermal titration calorimetry was performed on a VP-ITC microcalorimeter from MicroCal, Northampton, MA. As control, dilution experiments of the individual components (1N36S, Flavy, poly(allylamine)) were carried out. For the dye-dilution experiment, one initial injection of 15 μL to saturate the titration cell wall was followed by 35 injections of 7.5 μL each. The time span between subsequent injections was 100 s. All experiments were conducted at 25 °C. Data analysis was performed with the modified model described and implemented in the MicroCal ITC data analysis software for Origin 7.0.

## Supporting Information

File 1 ^13^C NMR assignments, UV–vis and light scattering data.
