# Supplementary material for: Multiswitchable photoacid–hydroxyflavylium–polyelectrolyte nano-assemblies
Source: Beilstein J Org Chem. 2021 Jan 19;17:166–85. doi: 10.3762/bjoc.17.17 (PMC7849232; doi:10.3762/bjoc.17.17)
Supplement: File 1 — 13C NMR assignments, UV–vis and light scattering data. [file Beilstein_J_Org_Chem-17-166-s001.pdf]

BEILSTEIN JOURNAL OF ORGANIC CHEMISTRY

Supporting Information

for

Multiswitchable photoacid–hydroxyflavylium–polyelectrolyte nano-assemblies

Alexander Zika and Franziska Gröhn

Beilstein J. Org. Chem. **2021,** *17,* 166–185. doi:10.3762/bjoc.17.17

^{13}C NMR assignments, UV–vis and light scattering data

License and Terms: This is a supporting information file under the terms of the Creative Commons Attribution License (https://creativecommons.org/licenses/by/4.0). Please note that the reuse, redistribution and reproduction in particular requires that the author(s) and source are credited and that individual graphics may be subject to special legal provisions.
The license is subject to the *Beilstein Journal of Organic Chemistry* terms and conditions: (https://www.beilstein-journals.org/bjoc/terms)

Table S1: ^{13}C NMR: Detailed signal assignment for different molecules. The color corresponds to the different molecules shown below.

Number of C-Atom	ppm
3	97.7
2	100.4
9	104.8
6	107.7
7	108.4
12	117.3
11	122.5
10	137.6
4	149.2
5	153.4
8	155.2
13	156.6
1	159.0
3/3	96.9
3	97.1
2	99.8
2/2	100.3
9	105.7
6/6	107.8
7/7	109.0
12/12	117.5
12	119.4
10	122.0
11	123.0
6	128.4
11	129.6
11	134.7
10	135.7
10	137.5
7	141.7
4/4/4	150.1
5	153.2
5	154.1
8/8	154.9
8	155.7
13/13/13	156.7
5	158.4
1/1/1	160.3
9	163.4
9	173.9

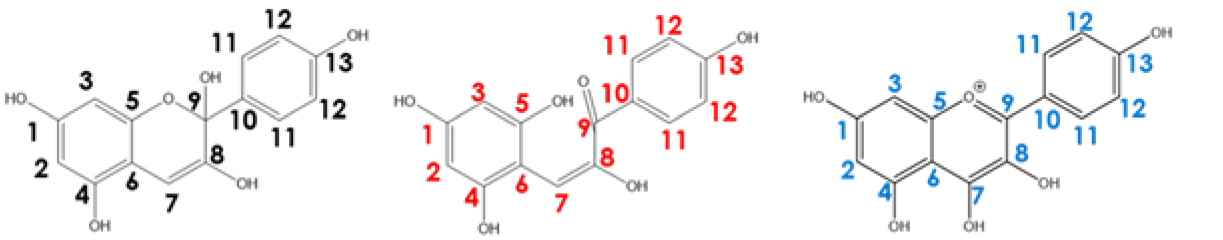

Scheme S1: Left: Flavy in form of ***B***. Middle: Flavy in form of ***Cc***. Right: Flavy in form of ***B₄***.

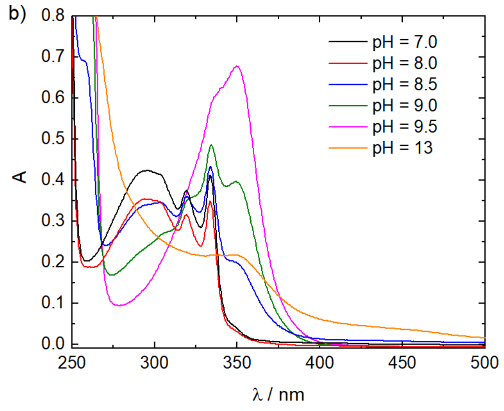

Figure S1: UV–vis of the photoacid 1N36S depending on the pH value of the solution.

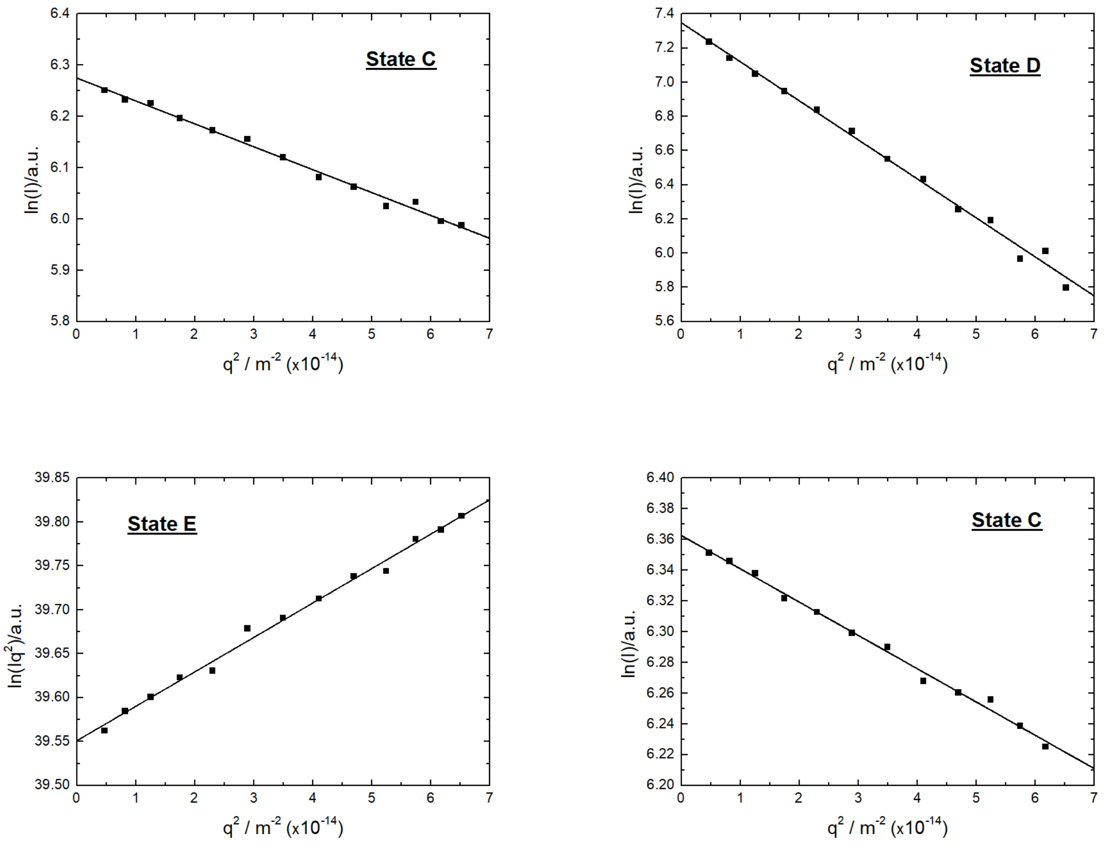

Figure S2: Static light scattering data from the assemblies of cycle II. Above left: **C**: pH 2.0. Above right: **D**: pH 5.0. Bottom left: **E**: Irradiation at λ = 300 nm. Bottom right: **C**: Dark and acidic environment.

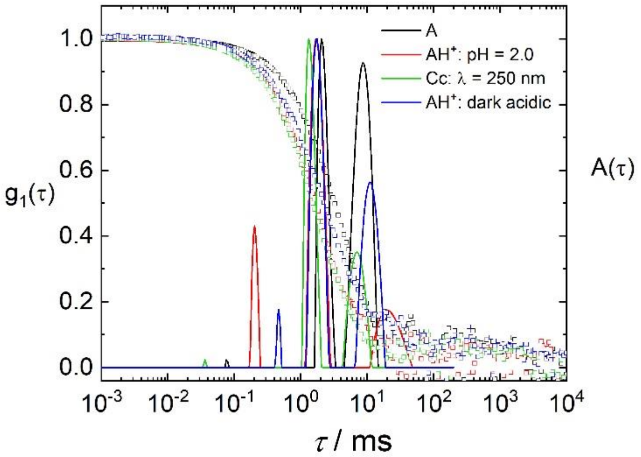

Figure S3: Electric field autocorrelation function $g_1(\tau)$ and distribution of relaxation times $A(\tau)$ at a scattering angle of Θ = 90° for the assemblies with the building blocks Flavy and PAA at a charge ratio of *l* = 0.5.

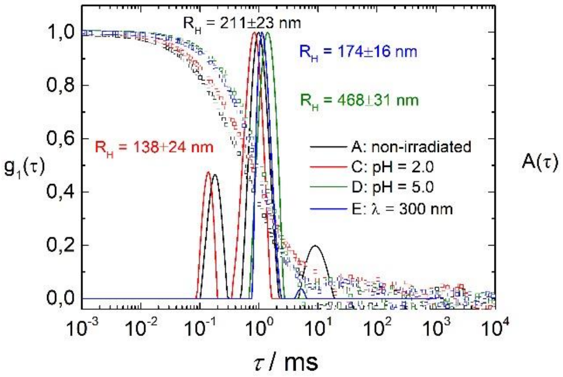

Figure S4: Electric field autocorrelation function $g_1(\tau)$ and distribution of relaxation times $A(\tau)$ at a scattering angle of Θ = 90° for the assemblies at a concentration ratio of l = 4.0.
